# Knowledge, attitudes and beliefs about vaccination in primary healthcare workers involved in the administration of systematic childhood vaccines, Barcelona, 2016/17

**DOI:** 10.2807/1560-7917.ES.2019.24.6.1800117

**Published:** 2019-02-07

**Authors:** Camila Andrea Picchio, Mireia Garcia Carrasco, Maria Sagué-Vilavella, Cristina Rius

**Affiliations:** 1 Servei Programes i Intervencions Preventives, Agencia de Salut Publica de Barcelona, Barcelona, Spain; 2 Servei d’Epidemiologia, Agencia de Salut Publica de Barcelona, Barcelona, Spain; 3 Universitat Pompeu Fabra, Barcelona, Spain; 4 Universitat Autonoma de Barcelona, Barcelona, Spain; 5 CIBER Epidemiología y Salud Pública (CIBERESP), Instituto Carlos III, Madrid, Spain

**Keywords:** vaccines, vaccine hesitancy, primary care, vaccine preventable diseases, infectious diseases, healthcare workers, questionnaire

## Abstract

**Background:**

Healthcare professionals are a reliable and impactful source of information on vaccination for parents and children.

**Objectives:**

We aimed to describe the knowledge, attitudes and beliefs primary care professionals involved in administration of childhood vaccines in Barcelona have about vaccines and vaccination.

**Methods:**

In 2016/17, surveys were administered in person to every public primary care centre (PCC) with a paediatrics department (n = 41). Paediatricians and paediatric nurses responded to questions about disease susceptibility, severity, vaccine effectiveness, vaccine safety, confidence in organisations, key immunisation beliefs, and how they vaccinate or would vaccinate their own children. We used standard descriptive analysis to examine the distribution of key outcome and predictor variables and performed bivariate and multivariate analysis.

**Results:**

Completed surveys were returned by 277 (81%) of 342 eligible participants. A quarter of the respondents reported doubts about at least one vaccine in the recommended childhood vaccination calendar. Those with vaccine doubts chose the response option ‘vaccine-hesitant’ for every single key vaccine belief, knowledge and social norm. Specific vaccine knowledge was lacking in up to 40% of respondents and responses regarding the human papilloma virus vaccine were associated with the highest degree of doubt. Being a nurse a risk factor for having vaccine doubts (adjusted odds ratio (ORa) = 2.0; 95% confidence interval (95% CI): 1.1–3.7) and having children was a predictor of lower risk (ORa = 0.5; 95% CI: 0.2–0.9).

**Conclusions:**

Despite high reported childhood immunisation rates in Barcelona, paediatricians and paediatric nurses in PCC had vaccine doubts, especially regarding the HPV vaccine.

## Introduction

The term vaccine hesitancy (VH) was defined in 2012 by the World Health Organization’s (WHO) Strategic Advisory Group of Experts (SAGE) on Immunization as ‘delay in acceptance or refusal of vaccination despite availability of vaccination services. Vaccine hesitancy is complex and context specific, varying across time, place and vaccines. It is influenced by factors such as complacency, convenience and confidence’ [1].

During the last decade, groups or subpopulations where vaccination coverage is below the required threshold because of VH have been associated with outbreaks and the reappearance of vaccine-preventable diseases, like measles [2]. In 2017, the WHO reported a total of 21,315 cases of measles and 35 deaths in the European Region of the WHO alone, representing an increase of 400% compared with the previous year [3]. In Barcelona, however, the situation was more stable despite 46 confirmed measles cases that originated from imported cases in the same year [4,5].

Notwithstanding the impact of the media and the easy access to the Internet, which can contribute positively or negatively [6] to the acceptance of childhood immunisation, healthcare professionals (HCPs) have repeatedly been identified as the most reliable and impactful source of information on vaccination for parents and their children [2,7-9]. Sixty-nine per cent of Spanish families identified their paediatrician as the most important source regarding vaccines [10]. Nonetheless, although it is internationally recommended [8] to work with this population to counter VH, HCPs in the region of Catalonia in Spain have not been studied.

Given that VH has been described among European vaccine providers [11], it is of the utmost importance to address the loss of confidence in vaccines in this population. Entire vaccination programmes could be jeopardised if healthcare professionals’ recommendations to immunise children are deficient as a result of VH [7].

Faced with this situation at the European level, and in spite of adequate vaccine coverage, the Public Health Agency of Barcelona (ASPB) launched in 2016 a line of research to monitor VH in HCPs and study its determinants in Barcelona. The main objective of this study was to describe the knowledge, attitudes and beliefs about vaccines among professionals who are directly involved in the administration of systematic childhood vaccines in the public health system of the city.

## Methods

This investigation is an observational cross-sectional study consisting of data collected through a structured survey.

### Population surveyed

In Barcelona, systematic childhood vaccination is recommended and administered by paediatric health professionals (paediatricians and paediatric nurses). The public health system covers more than 90% of all childhood vaccinations in the city. The study population enrolled were HCPs who were directly involved in the administration of systematic childhood vaccines in public primary care centres (PCC) in Barcelona. Family doctors or practitioners who specialise in fields not related to paediatrics and nurses who were not directly involved in the administration of childhood vaccines were excluded. Students, residents and temporary substitutes of any kind were also excluded.

Of the 54 PCC serving the city of Barcelona, 41 have a paediatrics department with overall 342 professionals.

### Questionnaire

A questionnaire was developed following available literature [12-14]. The questionnaire was translated into Spanish and Catalan and culturally adapted using the cognitive debriefing method [15]. Cognitive debriefing is a process where representatives of the target population actively test the translated questionnaires to determine whether respondents would understand the questionnaire as easily as the English version would be understood [16].

Respondents answered questions about disease susceptibility, disease severity, vaccine effectiveness, vaccine safety, who benefits from childhood immunisations, key immunisation beliefs, whether or not they had children, how they vaccinated or would vaccinate their own children, and whether they felt they had enough information and tools in order to adequately respond to vaccine-hesitant parents.

### Administration

Questionnaires were self-administered by the HCPs at the PCC during a date and time that was previously agreed between the investigators and contact person from each centre. Contact with centres began in March 2016 and ended in February 2017. Some centres were contacted up to six times.

### Variables

Demographical variables were collected. All other variable responses were categorised on a 5-point Likert scale that was later dichotomised. Responses to whether or not they would vaccinate their children against the listed vaccines included in the calendar were dichotomised into ‘yes’ and the outcome variable ‘vaccine doubts’ which was a combination of all other options (late/doubts/no). Disease susceptibility and severity variables were dichotomised into ‘likely/very likely’ vs all other responses and ‘severe/very severe’ vs all other responses. Vaccine safety was dichotomised into ‘safe/very safe/completely safe’ vs all other responses and vaccine efficacy was dichotomised into ‘protects/protects a lot’ vs all other responses. Benefit of vaccination was dichotomised into benefits ‘considerably/a lot’ vs all other responses. Key vaccine beliefs, knowledge and social norms were also dichotomised into the ‘vaccine-hesitant’ option vs no VH. Because the key affirmations were positive and negative, agreeing with the affirmation was the vaccine-hesitant option in some cases, while in others, disagreeing corresponded to VH. Missing values and don’t know/no response (DK/NR) were all treated the same in the analysis.

### Data analysis

Standard descriptive statistics was performed using STATA version 11.0. Mean, standard deviation and Student’s t-test were used for quantitative variables and frequency, chi-squared test, and Fisher’s exact test were used for qualitative variables. To study sociodemographic correlates with VH, a multiple logistic regression was performed where odds ratios (OR) were obtained with their 95% confidence intervals (95% CI), adjusting for all sociodemographic variables. Missing values and DK/NR were included only in the descriptive analysis. All analyses were based on two-sided p values with statistical significance defined by p ≤ 0.05. 

### Ethical considerations

This study was approved by the ethics committee of the Consorci Parc de Salut Mar de Barcelona. The researchers declare no conflicts of interest.

## Results

Of the 342 paediatric health professionals in the Barcelona public primary care centres, 277 (81%) participated in the study; 136 were paediatricians, 138 were paediatric nurses and three were not defined. The rate of participation of paediatricians and paediatric nurses was 76.8% and 83.6%, respectively. Only one PCC chose not to participate. The mean age was 48 years (SD = 10.5 years) and 244 (88.4%) were female. The mean number of years of experience was 23 years (SD = 10.5 years). Of those who responded to the survey, 75 (27.1%) reported not having children.

**Figure 1:**
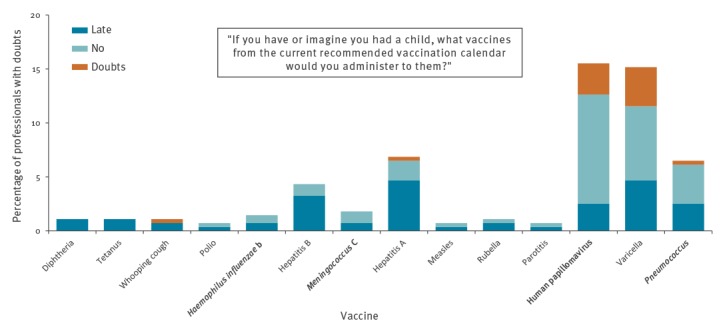
Paediatric health professionals who responded ‘late’, ‘doubts’, or ‘no’ to vaccinating their own children, survey about vaccine knowledge, attitudes and beliefs, Barcelona, 2016/17 (n = 277)

Of those that were surveyed, 71 (25.6%; 95% CI: 20.8–31.1) had doubts about at least one of the vaccines in the current vaccination calendar. Respondents reported the most doubt regarding the HPV and varicella vaccines (Figure 1, Supplementary Table S1). Excluding the HPV and varicella vaccines, 34 (12.3%; 95% CI: 8.8–16.7) of health professionals reported having a doubt about at least one vaccine in the current calendar.

Statistically significant differences existed between professions for the pneumococcus (3.8% vs 9.9%; p = 0.049), Hepatitis B (1.5% vs 7.6%; p = 0.034) and HPV (9.9% vs 22.9%; p = 0.005) vaccines where nurses reported more doubts. Statistically significant differences existed between those with and without children for the varicella vaccine (12.5% vs 25.4%; p = 0.012) where those without children reported more doubts.

### Perception of probability and severity of illness and of vaccine safety and protection

**Figure 2:**
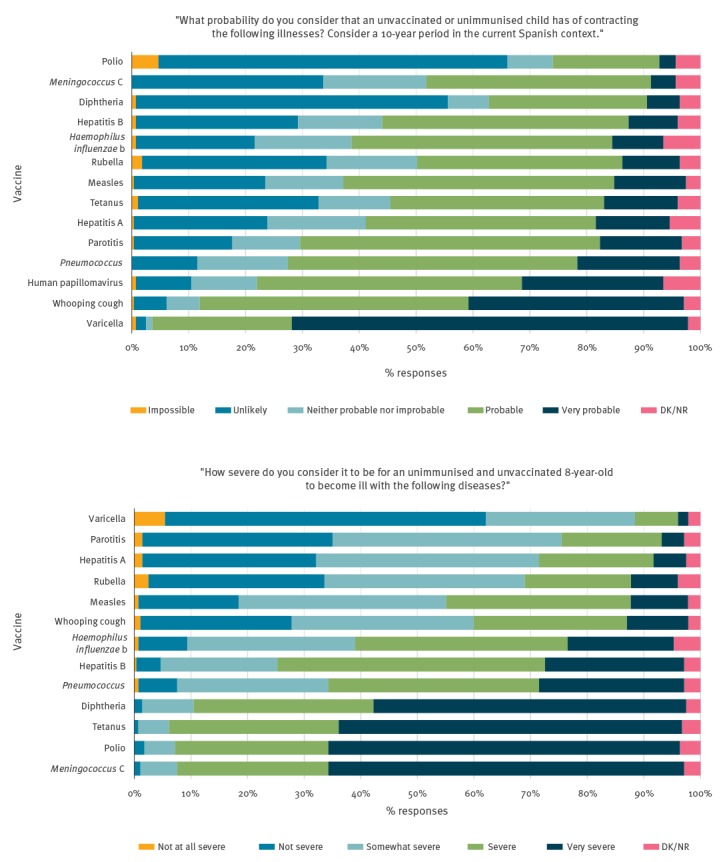
Disease susceptibility and severity perceived by paediatric health professionals, survey about vaccine knowledge, attitudes and beliefs, Barcelona, 2016/17 (n = 277)

**Figure 3:**
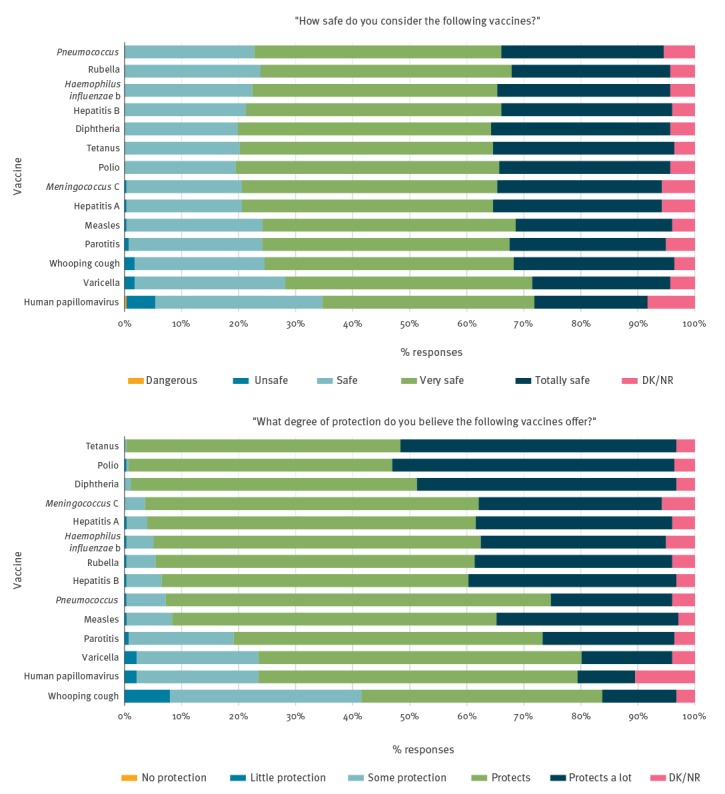
Vaccine safety and effectiveness perceived by paediatric health professionals, survey about vaccine knowledge, attitudes and beliefs, Barcelona, 2016/17 (n = 277)

Thirteen (4.7%) respondents felt that it would be impossible for an unvaccinated and unimmunised child to contract polio and 198 (71.5%) responded that it is probable or very probable for an unvaccinated child to contract HPV. Respondents reported that polio, illness from meningococcus C and tetanus were the most serious illnesses, with varicella being the least serious (Figure 2, Supplementary Tables S2 and S3). All vaccines were reported to be safe, with the exception of the HPV vaccine, which was described as dangerous by one participant and unsafe by 14 (5%). Five respondents also reported the varicella and whooping cough vaccines as being unsafe (Figure 3, Supplementary Tables S4 and S5). We saw the largest number of missing values for questions surrounding HPV. Eighteen missing values (6.5%) were received for HPV susceptibility, 23 (8.3%) for HPV vaccine safety and 29 (10.5%) for the level of protection the vaccine provides. In general, there were no statistically significant differences in sex, age or years of profession in relation to the variables on probability and severity of illness and on the protection offered by vaccines. Statistically significant differences were seen among those without children who reported more doubts regarding the safety of the HPV vaccine (11.5% vs 3%; p = 0.019). Statistically significant differences between professions were seen in almost every category.

DK: don’t know/NR: no response.

DK/NR: don’t know/no response.

### Key vaccine beliefs, knowledge and social norms

Of the 277 who participated, 269 (97.1%) believed that the child receiving the vaccine benefits considerably/benefits a lot from vaccination, 267 (96.4%) believed that the community benefits considerably/benefits a lot, 256 (92.4%) believed that health personnel benefit considerably/benefit a lot, 253 (91.3%) believed that the government benefits considerably/benefits a lot, and 244 (88.1%) believed that the pharmaceutical industry benefits considerably/benefits a lot. There were no significant differences regarding the responses to beliefs about the benefits of vaccination.

Of the 229 participants who believed that pharmaceutical companies benefit considerably/benefit a lot from vaccination and responded to the question about illegitimate interests influencing the vaccination calendar, 129 (56.3%) believed that the vaccines currently recommended are influenced by illegitimate pharmaceutical interests compared with 100 (43.7%) who did not believe this (p = 0.012).

**Figure 4:**
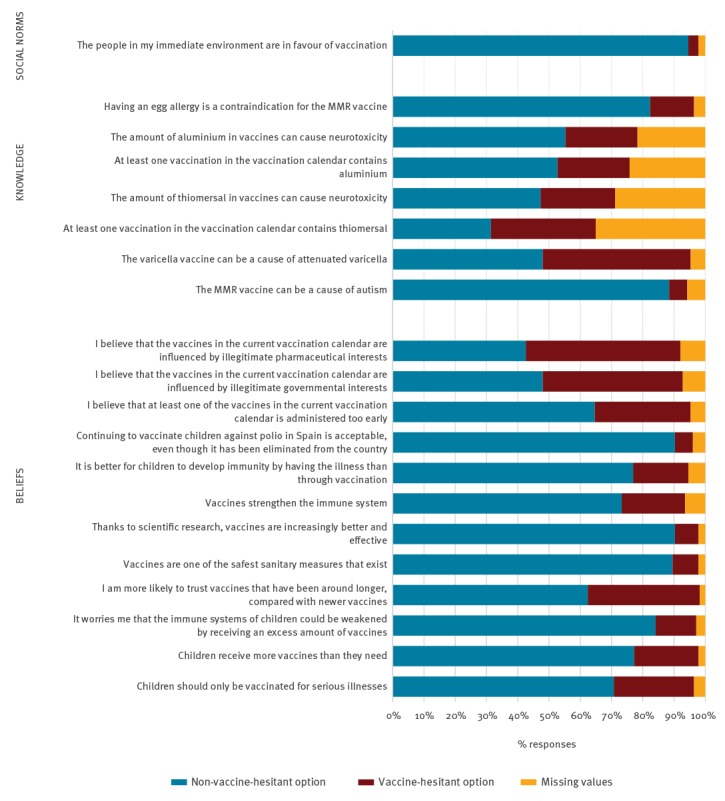
Key beliefs, knowledge and social norms about vaccines, by affirmation, survey among paediatric health professionals, Barcelona, 2016/17 (n = 277)

Twenty-five (12.8%) participants who reported having children felt worried that children’s immune systems could be weakened from receiving too many vaccines, and 65 (33%) of these same respondents believed that at least one vaccine in the current calendar is administered too early. A total of 262 (94.6%) participants reported that the people in their immediate environment were in favour of vaccination and 10 (3.6%) participants did not believe that thanks to scientific research, vaccines are increasingly better and more effective (Figure 4).

MMR: measles-mumps-rubella vaccine.

Responses to key vaccine knowledge, beliefs and social norms were categorised as either vaccine-hesitant or non-vaccine hesitant.

Key vaccine knowledge affirmations showed the highest number of missing values, and a higher percentage of respondents chose the VH option. A total of 133 (48.0%) respondents correctly responded that the varicella vaccine can cause attenuated varicella. With respect to the components that make up our vaccines today, 93 participants (33.5%) responded that at least one vaccine in the current vaccination calendar contains thiomersal and 97 (35%) did not know the answer or chose not to respond. Further, 80 respondents (28.9%) did not know or chose not to answer the question which stated that the amount of thiomersal in vaccines can cause neurotoxicity. In addition, 67 (24.2%) did not respond or did not know whether or not vaccines contain traces of aluminium, and 60 (21.7%) did not know whether or not the amount of aluminium in vaccines causes neurotoxicity (Figure 4).

Those with vaccine doubts chose the VH response option for every single key vaccine belief, knowledge and social norm (Table). Of those who had doubts about at least one vaccine in the current vaccination calendar, 60% responded that they believed that the current vaccines in the calendar were influenced by illegitimate governmental interests (p = 0.029). Similarly, of those who had vaccine doubts, 69% reported believing that the current vaccination calendar was influenced by illegitimate pharmaceutical interests (p = 0.004).

**Table:** Respondents with or without doubts about vaccines who selected the vaccine-hesitant option, survey among paediatric health professionals, Barcelona, 2016/17 (n = 277)

	Respondents with doubts (n = 71)^ a^		Respondents without doubts (n = 206)^a^		p value
	n	%	n	%	
Children should only be vaccinated for serious illnesses	36	53.7	35	17.50	< 0.001
Children receive more vaccines than they need	33	47.1	24	11.94	< 0.001
It worries me that the immune systems of children could be weakened by receiving an excess amount of vaccines	19	27.9	17	8.46	< 0.001
I am more likely to trust vaccines that have been around longer, compared with newer vaccines	33	47.1	66	32.67	0.030
Vaccines are one of the safest sanitary measures that exist	10	14.5	13	6.44	0.038
Thanks to scientific research, vaccines are increasingly better and effective	10	14.5	11	5.45	0.015
Vaccines strengthen the immune system	23	34.3	33	17.19	0.003
It is better for children to develop immunity by having the illness than through vaccination	24	36.3	25	12.76	< 0.001
Continuing to vaccinate children against polio in Spain is acceptable, even though it has been eliminated from the country	7	10.0	9	4.59	0.102
I believe that at least one of the vaccines in the current vaccination calendar is administered too early	36	54.5	49	24.75	< 0.001
I believe that the vaccines in the current vaccination calendar are influenced by illegitimate governmental interests	40	59.7	84	44.21	0.029
I believe that the vaccines in the current vaccination calendar are influenced by illegitimate pharmaceutical interests	46	68.6	91	48.40	0.004
The MMR vaccine can be a cause of autism	7	10.9	9	4.57	0.065
The varicella vaccine can be a cause of attenuated varicella	35	52.2	96	48.73	0.620
At least one vaccine in the vaccination calendar contains thiomersal	28	70.0	65	46.43	0.009
The amount of thiomersal in vaccines can cause neurotoxicity	17	37.7	49	32.24	0.489
At least one vaccine in the vaccination calendar contains aluminium	40	74.0	106	67.95	0.399
The amount of aluminium in vaccines can cause neurotoxicity	18	31.5	46	28.75	0.688
Having an egg allergy is a contraindication for the MMR vaccine	12	17.6	27	13.57	0.411
The people in my immediate environment are in favour of vaccination	6	8.4	3	1.50	0.011

MMR: measles-mumps-rubella.

^a^ Missing values were excluded.

Multivariate analysis adjusted for sociodemographic characteristics revealed the profession of nursing to be a risk factor for VH (ORa = 2.0; 95% CI: 1.1–3.7) and having children as a factor of less risk for VH (ORa = 0.5; 95% CI: 0.2–0.9).

### Professional practice and vaccine doubts

Overall, 81 (29.2%) of the 277 HCPs responded that they felt they did not have sufficient information and training to adequately answer questions vaccine-hesitant parents may have. The majority of respondents wished to receive more information about vaccines online 145 (52.3%) and through training sessions 139 (50.2%).

## Discussion

Our study showed that, in general, public paediatric professionals in Barcelona supported vaccination. However, one in four respondents reported having doubts about at least one vaccine in the current recommended childhood vaccination schedule. Half of the doubts expressed were described in association with the HPV and varicella vaccines. Moreover, we identified a lack of trust in the government and the pharmaceutical industry, a lack of knowledge about vaccine components and the belief in certain myths held by vaccine-hesitant parents.

Despite recent attempts in Spain to unify childhood vaccination schedules, different ones coexist: one for each autonomous region. The Catalan recommended vaccination calendar has been changed four times in the past 10 years [17]. The two vaccines which generated the most doubt in our study population were only recently added to the recommended systematic childhood vaccination calendar (HPV in 2008 and varicella in 2016), and their introduction was accompanied by social and scientific criticism. The doubts surrounding these vaccines described in our population can in part be explained by the frequent changes in the vaccination calendar and differing calendars within the country [18]. This lack of confidence in certain vaccines could be highlighting the need to improve communication between those who dictate public health policies and health professionals who directly care for families. Karafillakis et al. described similar scenarios in France and Greece where a lack of trust in the government and pharmaceutical industry could potentially stain the credibility of vaccine information [19].

While the vaccine effectiveness responses we received were in line with available literature [20-22], it is alarming that some health professionals considered vaccines that are being administered to children as unsafe or even dangerous. The large number of missing responses associated with the HPV vaccine indicates a sense of doubt or unawareness about this vaccine’s proven safety and efficacy. Karafillakis et al. also described that the HPV vaccine was singled out in their recent study and explain the hesitancy by the fact that it is a new vaccine [19].

Doubts surrounding the vaccine which protects young girls from HPV is in line with the opinions of certain groups [23], but indicating that the varicella, whooping cough and mumps vaccines are also unsafe, potentially indicates an inability to differentiate between vaccine safety and effectiveness, a crucial determinant when educating vaccine-hesitant parents.

Our respondents’ perceptions of disease severity and probability were almost identical to those reported by Salmon et al. [12]. In our study, however, the perception of probability of infection varied. Some professionals considered polio virus infection virtually impossible, an opinion that may have consequences. For example, overconfidence in the safety and effectiveness of a vaccine such as the one against polio, a disease assumed to be eliminated in our environment, could prevent an HCP from recommending the vaccine to families who have doubts about it. 

We interpret the high rate of missing values and DK/NR in our study as a gap in specific key vaccine knowledge. Hence, the conclusion can be drawn that HCPs administering vaccines to children in the public health system in Barcelona are lacking crucial information about vaccine components, contraindications and critical general vaccine knowledge. Paterson et al. described that overall, knowledge about particular vaccines, their efficacy and their safety helped build healthcare professionals’ own confidence in vaccines and their willingness to recommend them to others [9]. Improving vaccine knowledge among these professionals is crucial for guiding vaccine-hesitant parents and recommending vaccination.

The frequency of vaccine misconceptions in our study was similar to the study by Salmon et al. [12]. A large number of participants chose the VH response and did not answer questions related to myths that vaccine-hesitant parents might ask. Myths could become part of the belief structure of a society and our results suggest that the environment has already influenced the surveyed professionals in the same way as it does vaccine-hesitant families. Addressing this aspect would require working within the socio-cultural context as suggested by Yaqub et al. [2]. These investigators warned of the risks of focusing only on vaccine uptake rates and overlooking the underlying attitudes and beliefs associated with VH.

One third of those surveyed felt that they do not have sufficient information and training to adequately address questions from vaccine-hesitant parents. We must ensure that health professionals who are in contact with families are adequately informed and are capable of delivering clear and accurate messages to their patients [18,24]. To this effect, new and improved training workshops and information material need to be made available as continued education to these healthcare professionals as soon as possible. 

Our results must be interpreted in the context of several methodological limitations. While the high response rate is a strength of this study, it can also be seen as a drawback. The response rate, which surpasses those described in other similar studies [12,14,25], was achieved because the surveys were administered in person. This means that the self-reported evaluations may be subject to expectancy bias and complacency bias. In addition, there could have been a sample selection bias because the study examined only those in the public health sector who willingly participated. We are therefore unaware if those who did not wish to participate held more vaccine doubts or not. Nevertheless, we would like to emphasise that this is the first study that addresses this issue in our environment and that it was aimed at the entire population of paediatric primary care professionals in public centres in the city, which account for the majority of vaccinations in Barcelona.

## Conclusions

The data collected has proven useful for understanding VH in Barcelona and serves as a starting point for continued monitoring of VH in this large European city. Because differences among paediatricians and paediatric nurses were seen for almost every variable, and profession was the factor most associated with VH, a more detailed analysis by profession is currently underway.

In a time where other sources of information could potentially outweigh the importance of primary healthcare workers, it is crucial that those involved in the systematic administration of childhood vaccines are equipped with the skills and resources needed to manage the growing issue of VH.

## Supplementary Data

47000 Supplement
