# Supplementary material for: Knowledge, attitudes and beliefs about vaccination in primary healthcare workers involved in the administration of systematic childhood vaccines, Barcelona, 2016/17
Source: Euro Surveill. 2019 Feb 7;24(6):1800117. doi: 10.2807/1560-7917.ES.2019.24.6.1800117 (PMC6373069; doi:10.2807/1560-7917.ES.2019.24.6.1800117)
Supplement: Supplement [file 1800117_CARRASCO_Supplement.pdf]

Supplementary material

This supplementary material is hosted by *Eurosurveillance* as supporting information alongside the article *Knowledge, attitudes and beliefs about vaccination in primary healthcare workers involved in the administration of systematic childhood vaccines, Barcelona, 2016/17* on behalf of the authors who remain responsible for the accuracy and appropriateness of the content. The same standards for ethics, copyright, attributions and permissions as for the article apply. *Eurosurveillance* is not responsible for the maintenance of any links or email addresses provided therein.

Supplementary table S1.Peadiatric health professionals who responded 'late', 'doubts', or 'no' to vaccinating their own children, survey about vaccine knowledge, attitudes and beliefs, Barcelona, 2016/17 (n=277)

	**Late N (%)**	**No N (%)**	**Doubts N (%)**	**Missing values N (%)**
Diphtheria	3 (1.1)	0	0	10 (3.6)
Tetanus	3 (1.1)	0	0	9 (3.2)
Whooping cough	2 (0.7)	0	1 (0.4)	10 (3.6)
Polio	1 (0.4)	1 (0.4)	0	10 (3.6)
H. Influenzae b	2 (0.7)	2 (0.7)	0	11 (4.0)
Hepatitis B	9 (3.2)	3 (1.1)	0	12 (4.3)
Meningococcus C	2 (0.7)	3 (1.1)	0	10 (3.6)
Hepatitis A	13 (4.7)	5 (1.8)	1 (0.4)	9 (3.2)
Measles	1 (0.4)	1 (0.4)	0	10 (3.6)
Rubella	2 (0.7)	1 (0.4)	0	10 (3.6)
Parotitis	1 (0.4)	1 (0.4)	0	10 (3.6)
HPV	7 (2.5)	28 (10.1)	8 (2.9)	12 (4.3)
Varicella	13 (4.7)	19 (6.9)	10 (3.6)	13 (4.7)
Pneumococcus	7 (2.5)	10 (3.6)	1 (0.4)	9 (3.2)

Supplementary table S2. Disease susceptibility perceived by paediatric health professionals, survey about vaccine knowledge, attitudes and beliefs, Barcelona, 2016/17 (n = 277)

	**Impossible N (%)**	**Unlikely N (%)**	**Neither probable nor improbable N (%)**	**Probable N (%)**	**Very probable N (%)**	**DK/NR N (%)**
Diphtheria	2 (0.7)	152 (54.9)	20 (7.2)	77 (27.8)	16 (5.8)	10 (3.6)
Tetanus	3 (1.1)	88 (31.8)	35 (12.6)	104 (37.5)	36 (13.0)	11 (4.0)
Whooping cough	1 (0.4)	16 (5.8)	16 (5.8)	131 (47.3)	105 (37.9)	8 (2.9)
Polio	13 (4.7)	170 (61.4)	22 (7.9)	52 (18.8)	8 (2.9)	12 (4.3)
H. Influenzae b	2 (0.7)	58 (20.9)	47 (17.0)	127 (45.9)	25 (9.0)	18 (6.5)
Hepatitis B	2 (0.7)	79 (28.5)	41 (14.8)	120 (43.3)	24 (8.7)	11 (4.0)
Meningococcus C	0	93 (33.6)	50 (18.1)	109 (39.4)	12 (4.3)	12 (4.3)
Hepatitis A	1 (0.4)	65 (23.5)	48 (17.3)	112 (40.4)	36 (13.0)	15 (5.4)
Measles	1 (0.4)	64 (23.1)	38 (13.7)	132 (47.7)	35 (12.6)	7 (2.5)
Rubella	5 (1.8)	90 (32.5)	44 (15.9)	100 (36.1)	28 (10.1)	10 (3.6)
Parotitis	1 (0.4)	48 (17.3)	33 (11.9)	146 (52.7)	40 (14.4)	9 (3.3)
HPV	2 (0.7)	27 (9.7)	32 (11.5)	129 (46.6)	69 (24.9)	18 (6.5)
Varicella	2 (0.7)	5 (1.8)	3 (1.1)	68 (24.5)	193 (69.7)	6 (2.2)
Pneumococcus	0	32 (11.5)	44 (15.9)	141 (50.9)	50 (18.1)	10 (3.6)

DK/NR = Don't know/No Response

Supplementary table S3. Disease severity perceived by paediatric health professionals, survey about vaccine knowledge, attitudes and beliefs, Barcelona, 2016/17 (n =277)

	Not at all severe N (%)	Not severe N (%)	Somewhat severe N (%)	Severe N (%)	Very severe N (%)	DK/NR N (%)
Diphtheria	0	4 (1.4)	25 (9.0)	88 (31.8)	153 (55.2)	7 (2.5)
Tetanus	0	2 (0.7)	15 (5.4)	83 (30.0)	168 (60.6)	9 (3.2)
Whooping cough	3 (1.1)	74 (26.7)	89 (32.1)	75 (27.1)	30 (10.8)	6 (2.2)
Polio	0	5 (1.8)	15 (5.4)	75 (27.1)	172 (62.1)	10 (3.6)
H. Influenzae b	2 (0.7)	24 (8.7)	82 (29.6)	104 (37.5)	52 (18.8)	13 (4.7)
Hepatitis B	1 (0.4)	12 (4.3)	57 (20.6)	131 (47.3)	68 (24.5)	8 (2.9)
Meningococcus C	0	3 (1.1)	18 (6.5)	74 (26.7)	174 (62.8)	8 (2.9)
Hepatitis A	4 (1.4)	85 (30.7)	109 (39.4)	56 (20.2)	16 (5.8)	7 (2.5)
Measles	2 (0.7)	49 (17.7)	101 (36.5)	90 (32.5)	28 (10.1)	6 (2.2)
Rubella	7 (2.5)	86 (31.0)	98 (35.4)	52 (18.8)	23 (8.3)	11 (4.0)
Parotitis	4 (1.4)	93 (33.6)	112 (40.4)	49 (17.7)	11 (4.0)	8 (2.9)
Varicella	15 (5.4)	157 (56.7)	73 (26.4)	21 (7.6)	5 (1.8)	6 (2.2)
Pneumococcus	2 (0.7)	19 (6.9)	74 (26.7)	103 (37.2)	71 (25.6)	8 (2.9)

DK/NR = Don’t know/No Response

Supplementary table S4. Vaccine safety perceived by paediatric health professionals, survey about vaccine knowledge, attitudes and beliefs, Barcelona, 2016/17 (n=277)

	**Dangerous N (%)**	**Unsafe N (%)**	**Safe N (%)**	**Very safe N (%)**	**Totally safe N (%)**	**DK/NR N (%)**
Diphtheria	0	0	55 (19.9)	123 (44.4)	87 (31.4)	12 (4.3)
Tetanus	0	0	56 (20.2)	123 (44.4)	88 (31.8)	10 (3.6)
Whooping cough	0	5 (1.8)	63 (22.7)	121 (43.7)	78 (28.2)	10 (3.6)
Polio	0	0	54 (19.5)	128 (46.2)	83 (30.0)	12 (4.3)
H. Influenzae b	0	0	62 (22.4)	119 (43.0)	84 (30.3)	12 (4.3)
Hepatitis B	0	0	59 (21.3)	124 (44.8)	83 (30.0)	11 (4.0)
Meningococcus C	0	1 (0.4)	56 (20.2)	124 (44.8)	80 (28.9)	16 (5.8)
Hepatitis A	0	1 (0.4)	56 (20.2)	122 (44.0)	82 (29.6)	16 (5.8)
Measles	0	1 (0.4)	66 (23.8)	123 (44.4)	76 (27.4)	11 (4.0)
Rubella	0	0	66 (23.8)	122 (44.0)	77 (27.8)	12 (4.3)
Parotitis	0	2 (0.7)	65 (23.5)	120 (43.3)	76 (27.4)	14 (5.1)
HPV	1 (0.4)	14 (5.1)	81 (29.2)	103 (37.2)	55 (19.9)	23 (8.3)
Varicella	0	5 (1.8)	73 (26.4)	120 (43.3)	67 (24.2)	12 (4.3)
Pneumococcus	0	0	63 (22.7)	120 (43.3)	79 (28.5)	15 (5.4)

DK/NR = Don’t know/No Response

Supplementary table S5. Vaccine effectiveness perceived by paediatric health professionals, survey about vaccine knowledge, attitudes and beliefs, Barcelona, 2016/17 (n=277)

	No protection N (%)	Little protection N (%)	Some protection N (%)	Protects N (%)	Protects A lot N (%)	DK/NR N (%)
Diphtheria	0	0	3 (1.1)	139 (50.2)	126 (45.5)	9 (3.2)
Tetanus	0	0	1 (0.4)	133 (48.0)	134 (48.4)	9 (3.2)
Whooping cough	0	22 (7.9)	93 (33.6)	117 (42.2)	36 (13.0)	9 (3.2)
Polio	0	1 (0.4)	1 (0.4)	128 (46.2)	137 (49.5)	10 (3.6)
H. Influenzae b	0	1 (0.4)	13 (4.7)	159 (57.4)	90 (32.5)	14 (5.1)
Hepatitis B	0	1 (0.4)	17 (6.1)	149 (53.8)	101 (36.5)	9 (3.2)
Meningococcus C	0	0	10 (3.6)	162 (58.5)	89 (32.1)	16 (5.8)
Hepatitis A	0	1 (0.4)	10 (3.6)	159 (57.4)	95 (34.3)	11 (4.0)
Measles	0	1 (0.4)	22 (7.9)	157 (56.7)	88 (31.8)	8 (2.9)
Rubella	0	1 (0.4)	14 (5.1)	155 (56.0)	96 (34.7)	11 (4.0)
Parotitis	0	2 (0.7)	51 (18.4)	150 (54.2)	64 (23.1)	10 (3.6)
HPV	0	6 (2.2)	59 (21.3)	155 (56.0)	28 (10.1)	29 (10.5)
Varicella	0	6 (2.2)	59 (21.3)	157 (56.7)	44 (15.9)	11 (4.0)
Pneumococcus	0	1 (0.4)	19 (6.9)	187 (67.5)	59 (21.3)	11 (4.0)

DK/NR = Don't know/No response
